# The Influence of Powder Milling on Properties of SPS Compacted FeAl

**DOI:** 10.3390/molecules25092263

**Published:** 2020-05-11

**Authors:** Alena Michalcová, Murat Özkan, Pavol Mikula, Ivo Marek, Anna Knaislová, Jaromír Kopeček, Dalibor Vojtěch

**Affiliations:** 1 Departments of Metals and Corrosion Engineering, University of Chemistry and Technology, 166 28 Prague 6, Czech Republic; muratozkan473@hotmail.com (M.Ö.); mareki@vscht.cz (I.M.); knaisloa@vscht.cz (A.K.); vojtechd@vscht.cz (D.V.); 2 Nuclear Physics Institute ASCR, v.v.i., 250 68 Řež, Czech Republic; mikula@ujf.cas.cz; 3 FZU—Institute of Physics of the CAS, Na Slovance 1999/2, 182 21 Prague 8, Czech Republic; kopecek@fzu.cz

**Keywords:** FeAl, ball milling, SPS compaction, neutron diffraction

## Abstract

The Fe-28 at.% Al alloy was studied in this article. The aim was to describe the influence of gas atomized powder pre-milling before SPS (Spark Plasma Sintering) sintering on the structure and properties of the bulk materials. The initial powder was milled for 0.5, 1, and 8 h. It was proven that 1 h milling leads to the change in size and morphology of the particles, B2→A2 phase transformation, and to the contamination with the material from a milling vessel. Powder materials were compacted by the SPS process at 900, 1000, and 1100 °C. The differences between the bulk materials were tested by LM, SEM, and TEM microscopy, XRD, and neutron diffraction methods. It was proven that, although the structures of initial powder (B2) and milled powder (A2) were different, both provide after-sintering material with the same structure (D0_3_) with similar structural parameters. Higher hardness and improved ductility of the material sintered from the milled powder are likely caused by the change in chemical composition during the milling process.

## 1. Introduction

Fe-Al alloys are well-known for their many attractive properties such as low cost, low density, and good corrosion resistance [1,2,3,4,5]. The Fe-Al alloys with about 20–50 at.% Al are called iron aluminides. The Fe-Al alloys can be used for structural application at high temperatures because they have high temperature strength. This shows that it might replace stainless steels in some applications [6,7]. They are also potential materials for special uses such as fuel injector nozzles [8,9,10], automobile exhaust systems [11], and fuel filter elements in coal gasification systems [10].

The major issues in the mechanical properties of the Fe-Al alloys is their poor room temperature ductility. The solutions might be in the grain size refinement. It was proven that the Fe-Al alloy with a grain size less than 10 µm should be sufficiently ductile for industrial processing [12]. Material with this grain size is not obtainable by the conventional methods like casting and must be produced by advanced technologies consisting of two steps. In the first step, powder is prepared. High energy ball milling and atomization are among the most common methods for obtaining fine powder with fine grains [7,13]. The second step in obtaining bulk fine-grained materials is powder compaction. Several consolidation methods are available such as rapid sintering [14,15], spraying processes like high velocity oxy fuel (HVOF) [15], cold spraying [16], hot compression [17], hot isostatic pressing [18], detonation gun [19], additive manufacturing [20], and spark plasma sintering (SPS) [13,14,15,21,22,23,24,25,26,27,28].

Preparation of the Fe_3_Al–based materials by mechanical alloying form pure Fe and Al, which is followed by the SPS (spark plasma sintering) process already described [29,30,31]. The long-term milling process carried out in a stainless-steel vessel led to contamination of the milled material by carbon and consequent formation of carbides [30,31]. Their presence causes extreme brittleness of the material. To cut down the amount of contamination, the gas atomized powder was used instead of the pure elements in this work. The milling time needed to decrease the grain size is shorter than the one necessary for iron aluminides formation.

It was shown [32] that the Fe-28 at.% Al material prepared by melt atomization contained grains with an average size of 8 µm. Additive manufacturing of this powder led to production with a significantly larger grain size. According to the study on the Fe-43 at.% Al [33], the temperature of 1000 °C should be sufficient to obtain a compact material. In this work, the influence of sintering temperature and influence of pre-milling on the microstructure and properties of the bulk Fe-28 at.% Al materials (denoted as Fe28Al) were studied on the basis of X-ray diffraction, neutron diffraction, and microscopy.

## 2. Results and Discussion

Chemical composition of the initial Fe28Al was analyzed by X-Ray fluorescence spectroscopy (XRF) and the results are given in Table 1.

Table 1 shows the composition of powders after ball milling for different times. Contamination by material from a milling vessel (stainless steel) can be observed. The increase of content for powder milled for 0.5 and 1 h is up to 1 wt.% for all the impurity elements, but the change after 8 h of milling is significant. Comparing the contents of Ni and Cr, it can be seen that the contamination is not uniform for elements forming the stainless-steel vessel (18% Cr, 10% Ni). To prove that the high Cr content was not caused by an experimental error, the composition of SPS sintered material was also analyzed (see Table 1). The Cr contamination originating from the milling vessel have not been described yet. On the other hand, contamination by C is known [30,31].

**Figure 1:**
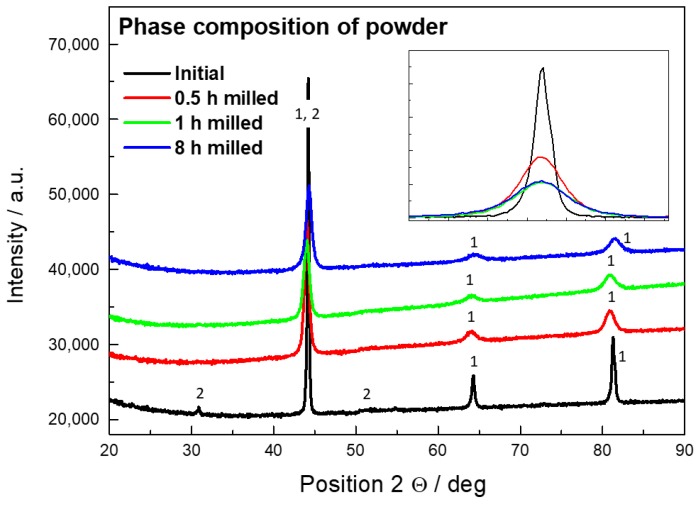
XRD pattern of Fe28Al powder in the initial state and after milling for 0.5, 1, and 8 h, 1—A2 FeAl, 2—B2 FeAl.

Figure 1 shows phase compositions of the initial and the milled powders. The initial powder has B2 phase composition (ordered FeAl), proven by the presence of the characteristic peak at 30.7 °. All the milled powders exhibit the A2 structure (disordered FeAl). The equilibrium composition of the Fe28Al at room temperature is Fe_3_Al in the D0_3_ structure. The transformation temperatures for a similar alloy were estimated to be 543 °C for D0_3_ ↔ B2 and 868 °C for B2 ↔ A2 [34,35]. The initial powder was processed by gas atomization and this technique is often used for rapid solidification. Because of this kinetic reason, the B2 structure was frozen to the room temperature. The ball-milling process involves high energy that is used for mechanical deformation but also partially for heating of the material. Due to this severe thermoplastic deformation, the Fe28Al transformed into the A2 phase.

The peaks from the 0.5-h milled powder exhibit less broadening than those from the 1-h milled powder, as can be seen in a detail window in Figure 1. The crystallite (coherently diffracting domain) sizes estimated by Scherrer calculator are given in Table 2. The difference in crystallite size between the 1-h and the 8-h milled powder was not significant, while the difference in contamination is huge. For further work, the powder milled for 1 h was chosen.

**Figure 2:**
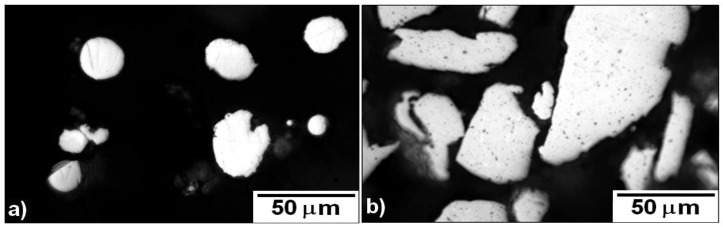
Optical micrographs of Fe28Al powder particles: (**a**) original and (**b**) 1-h milled.

**Figure 3:**
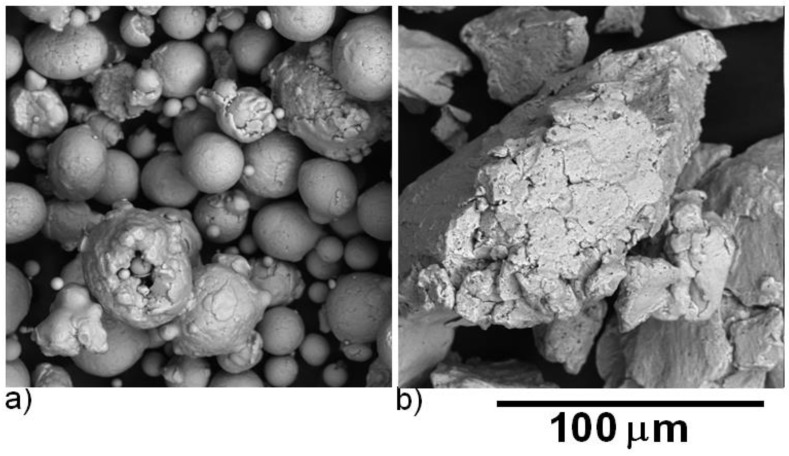
SEM (BSE) micrographs of Fe28Al powder particles: (**a**) original and (**b**) 1-h milled.

The evolution of morphology of powder particles after 1 h milling is shown in Figure 2 and Figure 3. The particles of the initial powder had a spherical shape, while the particles of the 1-h milled powder were irregular. The particle size estimated from metallographic samples was 18 ± 10 µm for the initial powder (in agreement with used granulometric fraction ≤ 45 µm) and 58 ± 28 µm for the 1-h milled powder.

Detailed observation by SEM confirmed changes in the morphology of powder particles. The initial powder is formed of spherical particles that are partially clustered as a consequence of the atomization process. The 1-h milled powder particles have irregular shape originating in welding and crushing of the initial spherical particles.

**Figure 4:**
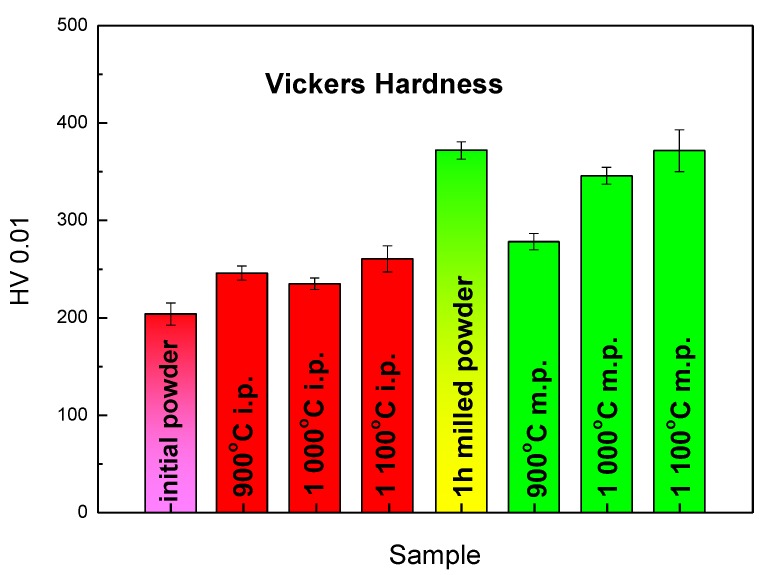
Hardness HV 0.01 of initials and 1-h milled Fe28Al powder and of SPS compacted Fe28Al from the initial powder sintered at 900 °C, 1000 °C, 1100 °C, and from 1-h milled powder sintered at 900 °C, 1000 °C, and 1100 °C.

The ball milling process leads to the increase of materials hardness (see Figure 4). For the comparison of the initial and the 1-h milled powder, a simple explanation by mechanical strengthening might be sufficient. However, there are also other aspects for the hardness changes including the grain size refinement and the phase transformation (B2→A2).

**Figure 5:**
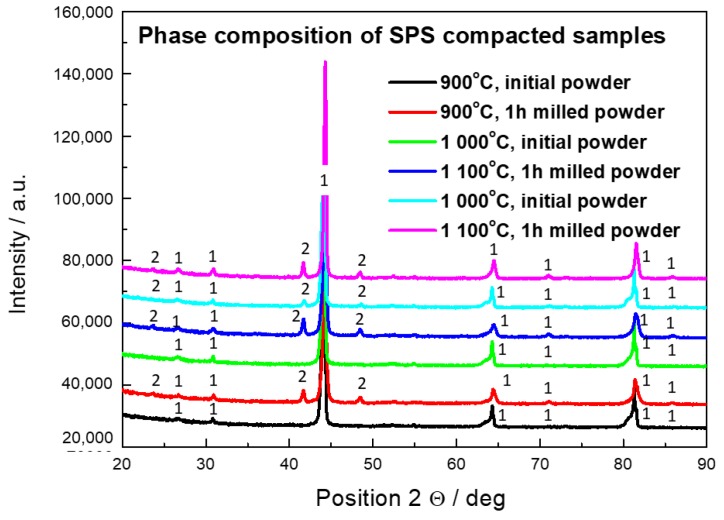
XRD diffraction pattern of SPS compacted Fe28Al from initial powder sintered at 900 °C, 1000 °C, and 1100 °C and from 1-h milled powder sintered at 900 °C, 1000 °C, and 1100 °C, 1—D0_3_ FeAl, 2—Fe_3_AlC_0.5._

The increase of hardness in case of the SPS compacted bulk materials from the initial powder is caused due to B2→D0_3_ phase transformation [36]. As shown in Figure 5, all the bulk materials are formed mainly by the D0_3_ phase (Fe_3_Al). The effect of phase transformation even compensated the expected decrease of hardness due to the grain coarsening during the compaction process.

The bulk material sintered from the initial powder at the highest temperature (1100 °C) contained a small amount of a Fe_3_AlC_0.5_ carbide. The energy of Fe_3_AlC_0.5_ carbide formation equals 120 KJ/mol compared to 90 KJ/mol for Fe_3_Al [37]. This might be the reason why the carbides were not detected in the materials sintered at a lower temperature. The sources of the carbon are the die and the protection foil both form by graphite, used in the SPS process. The elevated temperature during the SPS process enables carbon diffusion. The effect of A2 ↔ B2 phase transformation on diffusion of alloying elements in the matrix is negligible [38]. Based on this fact, it is possible to extrapolate the diffusion coefficients given in Reference [39] to the temperatures of SPS conditions. The carbon penetration depths calculated according to the parabolic law are 77 nm for 900 °C, 123 nm for 1000 °C, and 190 nm for 1100 °C. These amounts are under the detection of XRD, which implies that there is another method of carbon contamination than solid state diffusion. The carbon uptake during the sintering process is influenced by the used heating rate [40]. The parabolic law seems to be valid up to a heating rate of 10 K/s. The heating rate of 100 K/s caused evaporation of carbon and its diffusion in a gas state in the pores [40]. The penetration depth in this case is significantly material dependent. It was reported that spinel sintered with a heating rate of 100 K/s contained carbon contamination in the whole sample [41], while Sm sintered under the same conditions was contaminated to the depth of 10 µm [42].

The amounts of the carbide in the bulk samples sintered from the 1-h milled powder are higher. The reason is contamination of the milled powder by carbon, which is not shown in Table 1 since carbon cannot be analyzed by XRF. On the other hand, the content of carbon in the stainless steel is less than 1%. The more probable explanation is that the milled powder contains more pores and defects, which enables faster diffusion. The Fe_3_AlC_0.5_ peaks (at 41.8° and 48.4°) are less pronounced for the material sintered at 900 °C. This is in an agreement with the observation made for the materials sintered from the initial powder. At this temperature, the diffusion of carbon from the die and the foil is not significant and all the carbide content originates from the carbon that came to the material as a form of contamination during milling. The energy of carbide formation may again play its role. The hardness of the bulk material sintered from the milled powder at 900 °C is also very close to the values obtained from the materials sintered from the initial powder. The hardness values of materials sintered from the milled powder and the content of the Fe_3_AlC_0_ at 1000 and 1100 °C were higher. It was proven that the presence of the in-situ formed carbides leads to the higher hardness of the material [43].

**Figure 6:**
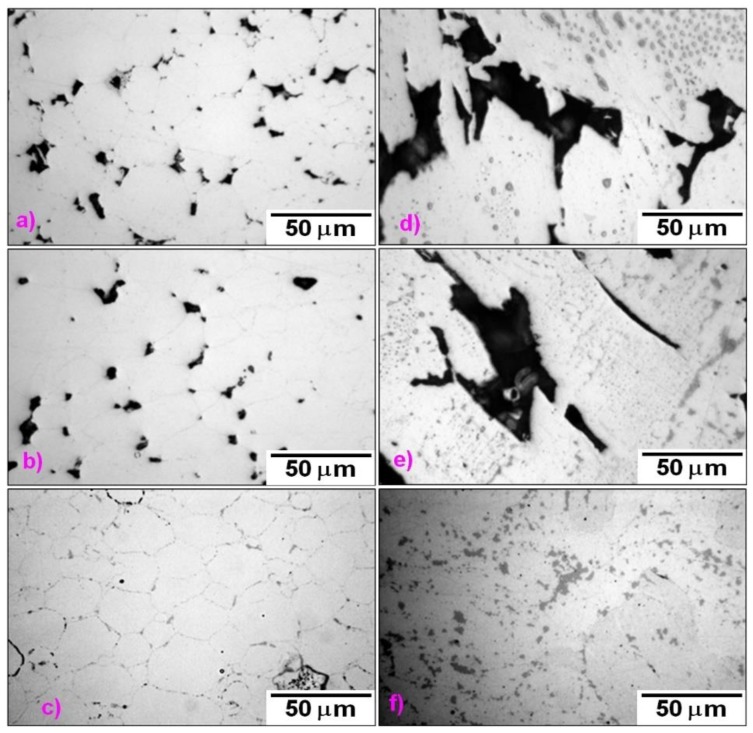
Optical micrographs of SPS compacted Fe28Al from the initial powder sintered at (**a**) 900 °C, (**c**) 1000 °C, and (**e**) 1100 °C and from 1-h milled powder sintered at (**b**) 900 °C, (**d**) 1000 °C, and (**f**) 1100 °C.

Microstructures of the bulk materials are shown in Figure 6. Sintering at 900 °C leads to compact material with a high amount of residual porosity for both powder materials (see Table 3). The materials sintered at a temperature of 1000 °C also exhibited a high amount of residual porosity, but it is clear that the particles were sintered into larger islands of a compact material. Sintering at the highest temperature—1100 °C—leads to the formation of a bulk material with a negligible residual porosity in both cases. In the materials sintered from the milled powder, contamination by carbides is visible as black spots. In the material sintered from the initial powder at 1100 °C, the boundaries of the individual powder particles are still visible, which might be caused by a thin oxide layer on the surface of the initial powder particles.

**Figure 7:**
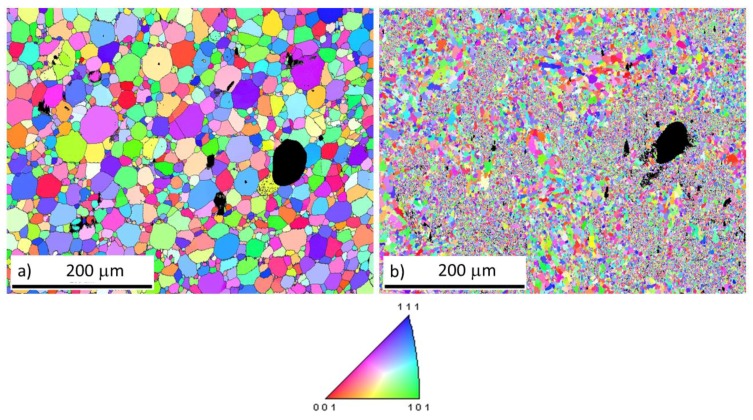
EBSD orientation maps of Fe28Al SPS compacted at 1100 °C sintered from (**a**) the initial and (**b**) the 1-h milled powder.

The EBSD orientation maps were measured on the TEM samples, which explains the black holes in both materials in Figure 7. The difference between the materials sintered at 1100 °C is remarkable. The material sintered from the initial powder is formed by polyhedral grains with a low grain-size distribution. On the other hand, the material sintered from the 1-h milled powder exhibited an extremely fine structure with bimodal distribution. Areas with nanometer-sized grains and areas with micrometer-sized grains. A similar structure was observed in Fe_3_Al material with 2 at% [44].

The results in Table 3 show porosity measured by image analysis and crystallite size determined by the Scherrer calculator. The grain size is obtained from EBSD data. The crystallite size is not very reliable since all the broadening of the XRD peaks was granted only to the grain size and the residual stresses were omitted.

**Figure 8:**
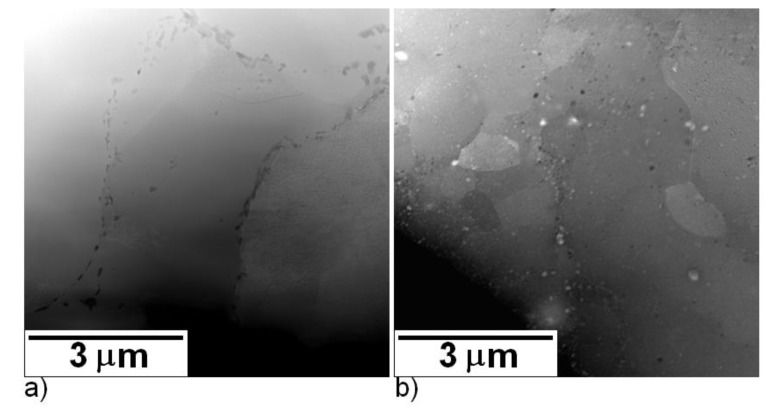
STEM/HAADF micrographs of Fe28Al spark plasma sintering (SPS) compacted at 1100 °C sintered from (**a**) initial powder and (**b**) 1-h milled powder.

A detailed microstructural observation carried out by TEM of the materials sintered at 1100 °C is given in Figure 8. Figure 8a shows the microstructure of the SPS compacted material sintered from the initial powder. Dark spots are oxide particles along the boundaries of the initial powder particles. The oxide particles do not form a homogeneous layer, which enables successful compaction of the material. Grains are visible inside of the sintered particles distinguishable by a different type of boundary. At the grains’ boundaries, the oxide particles are not present. From the limited field of view of TEM, the grains shown in Figure 8a seem to be about 2–3 µm in size.

Figure 8b documents the microstructure of the material sintered from the 1-h milled powder at 1100 °C. It can be seen that the material contains a significant number of heterogeneous particles both heavier and lighter than Fe28Al matrix (oxides, carbides). The grain size of the material sintered from the 1-h milled powder is finer, at about 2-µm in size (from the TEM micrographs).

**Figure 9:**
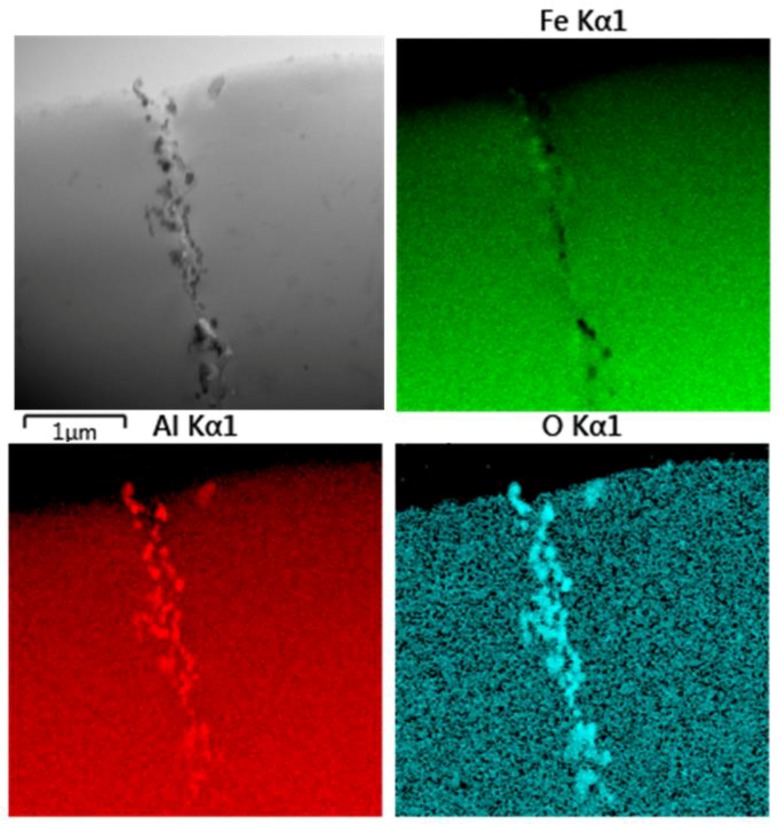
STEM/BF detailed micrographs and EDS elemental maps of Fe28Al SPS compacted at 1100 °C sintered from the initial powder.

Figure 9 shows the microstructure and the EDS elemental maps taken on the boundary of the initial powder particles in the materials sintered at 1100 °C from the initial powder. It can be seen that the boundary is decorated by fine oxide particles.

**Figure 10:**
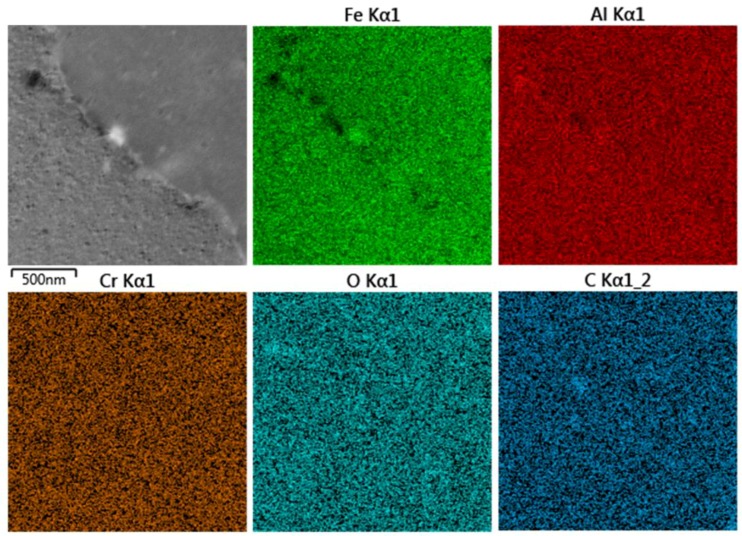
STEM/BF detailed micrographs and EDS elemental maps of Fe28Al SPS compacted at 1100 °C sintered from 1-h milled powder.

Figure 10 shows the microstructure and the EDS elemental maps in the materials sintered at 1100 °C from the 1-h milled powder. One carbide particle can be seen in the micrograph and Fe, Al, and C elemental maps. The Cr distribution is uniform, which is caused be good diffusion of Cr in the Fe_3_Al matrix [45] even when the Cr originates from the stainless steel [46].

Based on the results shown above, two main hypotheses about the difference in the hardness of the sintered materials can be discussed. The material sintered from the milled powder is strengthened by both dispersoids and the different phase transformation when compared with the material sintered from the initial powder. Due to its large interaction volume, a high-resolution neutron diffraction was performed in order to determine some differences in the structure of the matrix. The patterns obtained by the neutron diffraction contain information from the whole analyzed sample and not only from the surface layer, as it would be for the XRD.

**Figure 11:**
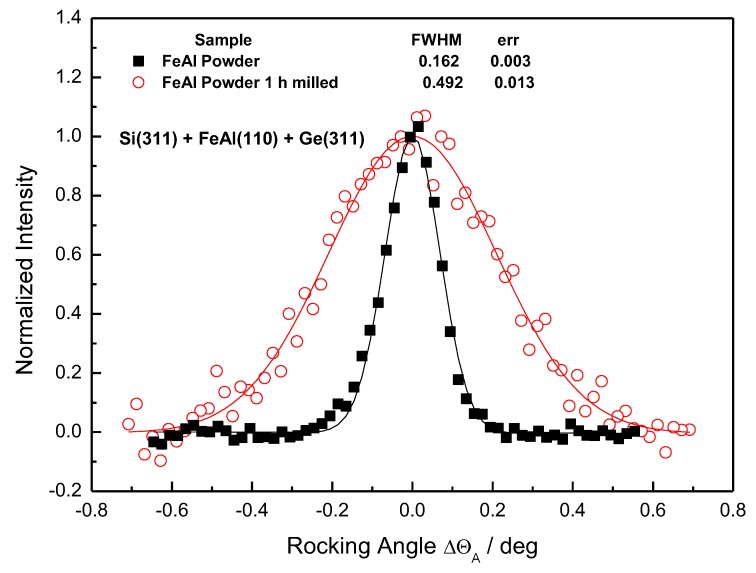
Analyzer rocking curves for the Fe28Al initial powder and the Fe28Al 1-h milled powder.

**Figure 12:**
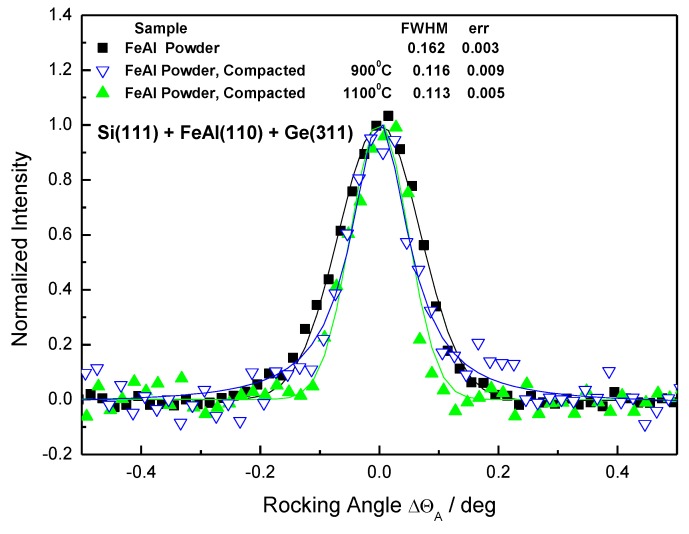
Analyzer rocking curves for the SPS compacted Fe28Al from the initial powder sintered at 900 °C, 1000 °C, and 1100 °C.

**Figure 13:**
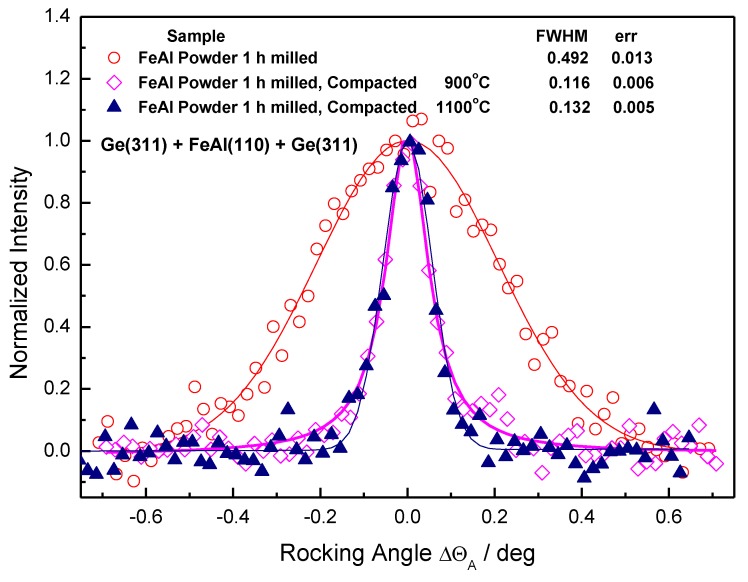
Analyzer rocking curves for the SPS compacted Fe28Al from 1-h milled powder sintered at 900 °C, 1000 °C, and 1100 °C.

Figure 11, Figure 12 and Figure 13 shows the analyzer rocking curves for the FeAl samples. Figure 11 shows the effect of the ball milling on FWHM (full weight in half maximum) of the analyzer rocking curves for the initial powder sample and the 1-h milled one. It can be seen from Figure 11 that the effect is remarkable. The conversion of the Δθ_A_ angles to Δd_S_/d_S_ provides the values of FWHM(Δd_S_/d_S_) of 3.18 × 10^−3^ and 9.80 × 10^−3^ for the Fe28Al initial and the Fe28Al milled samples, respectively.

Subsequently, the SPS compacted samples were investigated and the related results are shown in Figure 12 and Figure 13. Although the peak shapes of both powders were significantly different, the shapes of all sintered materials—independently of the pre-processing of the powder and sintering temperature—were similar. Since the samples were of different geometries, the curves were normalized to the zero position on the x-label. Because of this reason, the individual shifts of the peak position brought about by a possible change of the mean value of the lattice constant Δd_S_ could not be evaluated.

Figure 13 shows a comparison of the 1-h milled powder and the bulk material sintered from it. The FWHM of the rocking curve from the bulk material sintered at 900 °C is within the experimental error, which is similar to the curves obtained from the materials sintered from the initial powder.

The rocking curve from the bulk material sintered at 1100 °C is slightly broader than the others obtained from bulk materials. This is most likely a mixed effect of the phase transformation and recrystallization kinetics and precipitation of oxides and carbides. Due to the accumulated stress in the 1-h milled powder, it is not possible to direct the diffraction line broadening to the rise of precipitates directly. The other factor is that SPS heating is fast and it is equivocal with the structure of the Fe28Al present during sintering.

To summarize neutron diffraction results, the milling brings about plastic deformation of the FeAl particles with a large distribution of the lattice spacing d_S_, which results in a large FWHM of the rocking curve. On the other hand, the sintering at the high temperature works as annealing, which makes the rocking curves much narrower with FWHM close to the one corresponding to the experimental resolution of the diffractometer setting with the standard α-Fe(110) sample (approximately 0.1 deg).

**Figure 14:**
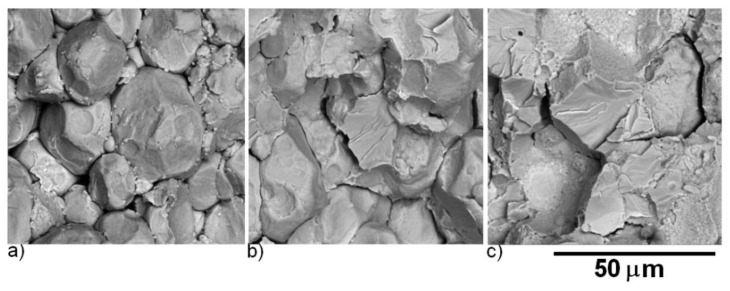
SEM (BSE) micrographs of fracture surfaces of SPS compacted Fe28Al from the initial powder sintered at (**a**) 900 °C, (**b**) 1000 °C, and (**c**) 1100 °C.

After the description of the microstructure, it was necessary to evaluate the success of the sintering process. Figure 14 shows the fracture surfaces after an impact test of the SPS compacted Fe28Al from the initial powder sintered at 900 °C, 1000 °C, and 1100 °C. The material sintered at 900 °C breaks completely along the surfaces of the initial particles. With an increasing sintering temperature, the fraction of inter-particular fracture is increasing even for material not fully inter-particular, but sintered at 1100 °C. This surface exhibits a brittle fracture with a river pattern.

**Figure 15:**
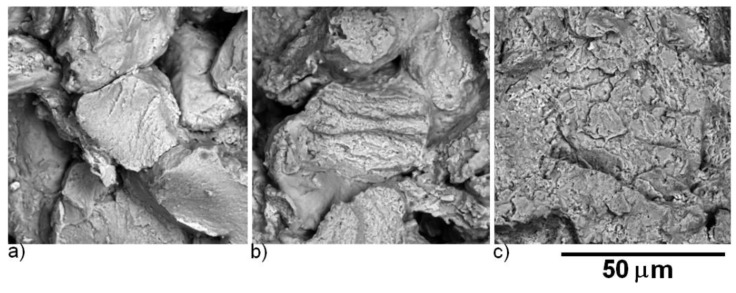
SEM (BSE) micrographs of fracture surfaces of the SPS compacted Fe28Al from the 1-h milled powder sintered at (**a**) 900 °C, (**b**) 1000 °C, and (**c**) 1100 °C.

As shown in Figure 15, the fracture surface of the material sintered at 900 °C from the 1-h milled powder is similar to the material sintered at the same temperature from the initial powder. The material sintered at 1000 °C exhibited a mixed fracture. The inter-particular fracture surface had a cup and cone pattern typical for a ductile fracture. This type of facture surface was observed within the whole sample sintered at 1100 °C from the 1-h milled powder.

It has already been found that the addition of 2–6 at.% of Cr leads to improving the ductility of the Fe28Al alloy [13]. The 1-h milled alloy contained 0.9 wt.% of Cr, which corresponds to 0.9 at.% in the actual composition. It is highly probable that the change in the fracture mechanism is connected with the Cr contamination.

## 3. Materials and Methods

In this work, the powder Fe-28 al.% Al (16 wt.%) prepared by nitrogen gas atomization was used. The powder was 0.5, 1, and 8 h milled using a planetary ball mill Retsch PM 100 (Retsch GmbH, Haan, Germany). For each batch, 5 g of the powder was taken and the ball-to-powder weight ratio of 10:1 at 400 rpm. The Ar protective atmosphere was used. The milling vessel and the milling balls (with a diameter of 1.1 cm, 10 balls were used) were made from stainless steel (18/10).

The initial and the 1-h milled powders were compacted using the FCT HP D 10 SPS machine (FCT Systeme HP D 10, Rauenstein, Germany). The SPS process was performed for 5 min under the Ar protective atmosphere at temperatures of 900, 1000, and 1100 °C. The graphite die was used with the separating graphite foils. The heating regime in the SPS was used. Ramping at the intended temperature with a heating rate of 200 K/min was followed by simultaneously applying a uniaxial force of 15 kN (48 mPa) and heat. The amount of 10 g was used for every compaction, which resulted in a cylindrical sample with a 19-mm diameter and a height between 4.5 and 5.5 mm.

The hardness of the samples was measured using the Future-Tech FM-700 device (Future-Tech Corp, Tokyo, Japan). The hardness was measured on embedded metallographic samples. At least 10 indents were measured using a load of 10 g with a 10-s dwell time.

The powders and the bulk samples were observed by Olympus PME3 optical microscope (Tokyo, Japan), by TESCAN VEGA 3LMU (20 kV accelerating voltage) (Brno, The Czech Republic) scanning electron microscope, TESCAN FERA 3 (EBSD mapping, 15 kV accelerating voltage, step 0.8 µm) (Brno, The Czech Republic) and by Jeol 2200 FS transmission electron microscope (200 kV accelerating voltage) (Jeol, Akishima, Japan). The TEM samples were prepared by ion polishing using the Gatan PIPs (Precision Ion Polishing system) (Gatan, Pleasanton, CA, USA).equipment.

The chemical composition was measured by XRF analysis was performed by ARL 9400 XP (XRF, ARL 9400 XP, Thermo ARL, Switzerland). The phase composition was analyzed by XRD using PANanalytical X’Pert PRO spectrometer (Panalytical, Almelo, The Netherlands).

Porosity was evaluated from optical micrographs using Image J 1.37v software (Wisconsin, WI, USA).

**Figure 16:**
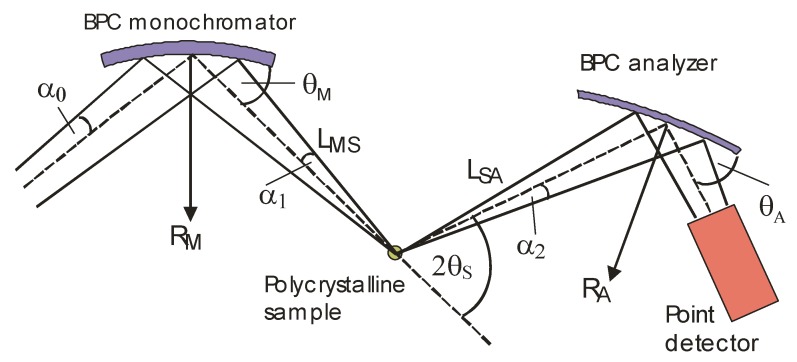
Schematic drawing of the three-axis diffractometer setting employing a BPC monochromator and analyzer (as used in the experiment, R_M_, R_A_—radii of curvature, θ_M_, θ_A_—Bragg angles) and operating at the neutron wavelength of λ = 0.162 nm.

To obtain an additional microstructural information, the unconventional high-resolution neutron diffraction setting was exploited [47,48]. Figure 16 shows the schematic drawing of the three axis neutron optics diffractometer (installed at the Řež medium power research reactor LVR-15) (Řež, Czech Republic), which was used for the experiment. By rocking the optimally curved analyzer, this setting is suitable for detail studies of individual diffraction lines. The studied Fe28Al samples were in the form of small plates of the dimensions of about 10 × 4 × 2 mm^3^ (length × width × thickness) and inserted in the neutron beam in the vertical position. Since the samples were not precisely of the same dimensions, the irradiated volumes were slightly different and, therefore, we could not compare the detector signal related to individual samples.

## 4. Conclusions

The initial Fe28Al powder was milled for 0.5, 1, and 8 h. It was proven that the milling leads to B2 → A2 phase transformation and to a contamination dependence on the milling time. The initial powder and the 1-h milled powder were compacted by SPS at 900, 1000, and 1100 °C. The materials sintered at 900 °C exhibited fully brittle fracture behavior in both cases. The fracture surface of the material sintered from the initial powder at 1100 °C was mixed between brittle and ductile. On the other hand, the behavior of the material sintered from the 1 h milled powder at 1100 °C was ductile. The difference between these materials was tested by the microscopy as well as the XRD and the neutron diffraction methods. It was proven that, although the structure of the initial powder (B2) and the milled powder (A2) were different, both provided the material with the same structure (D0_3_), which had similar structural parameters. The difference in properties (higher hardness) and behavior (ductility) was caused by the change in chemical composition during the milling process.

## Figures and Tables

**Table 1:** Chemical composition of Fe28Al powder in the initial state and after milling for 0.5, 1, and 8 h measured by XRF.

Element	Initial Powder	0.5 h Milled	1 h Milled	8 h Milled	Comp. Initial	Comp. 8 h Milled
Fe	83.34 ± 0.10	88.44 ± 0.20	86.90 ± 0.20	86.28 ± 0.20	86.10 ± 0.50	83.70 ± 0.60
Al	16.50 ± 0.08	10.34 ± 0.09	11.50 ± 0.10	4.58 ± 0.06	13.66 ± 0.10	8.23 ± 0.10
Cr	0.02 ± 0.01	0.61 ± 0.02	0.93 ± 0.03	6.18 ± 0.03	0.04 ± 0.01	5.47 ± 0.10
Mn	0.04 ± 0.01	0.08 ± 0.01	0.10 ± 0.01	0.73 ± 0.02	0.06 ± 0.01	0.34 ± 0.03
Si	0.10 ± 0.01	0.32 ± 0.02	0.52 ± 0.02	1.21 ± 0.03	0.14 ± 0.01	1.62 ± 0.05
Ni	n.d.	0.06 ± 0.01	0.04 ± 0.01	0.48 ± 0.02	n.d.	0.36 ± 0.06
Co	n.d.	0.16 ± 0.01	n.d.	0.54 ± 0.02	n.d.	0.28 ± 0.03

“n.d.” - not detected. The results are given in wt.%.

**Table 2:** Crystallite size of Fe28Al powder in the initial state and after milling for 0.5, 1, and 8 h measured by the Scherrer calculator from XRD patterns, given in nm.

Material	Initial Powder	0.5 h Milled	1 h Milled	8 h Milled
Crystallite size	392 ± 29	160 ± 5	135 ± 29	124 ± 28

**Table 3:** Porosity, crystallite size, and grain size of materials sintered at 900, 1000, and 1100 °C from Fe28Al powder in the initial state and after 1 h of milling. The crystallite size determined by Scherrer calculator. The grain size obtained from EBSD data.

SPS Temperature °C	Powder	Porosity %	Crystallite Size nm	Grain Size µm
900	Initial	5.2 ± 0.5	410 ± 56	-
1000	Initial	3.9 ± 0.4	501 ± 83	-
1100	Initial	0.5 ± 0.4	562 ± 89	18.6 ± 9.8
900	1 h milled	18.7 ± 1.3	323 ± 13	-
1000	1 h milled	14.2 ± 0.9	286 ± 62	-
1100	1 h milled	0.4 ± 0.2	561 ± 141	5.9 ± 3.0
